# Positive geographic correlation between soldiers’ weapon size and defensive prowess in a eusocial aphid, *Ceratovacuna japonica*

**DOI:** 10.1038/s41598-022-20389-z

**Published:** 2022-09-23

**Authors:** Mitsuru Hattori, Masato Ono, Takao Itino

**Affiliations:** 1 Graduate School of Fisheries and Environmental Sciences, Nagasaki University, Nagasaki City, Nagasaki 852-8521 Japan; 2 Division of Applied Entomology and Zoology, Graduate School of Agriculture, Tamagawa University, Machida, Tokyo 194-8610 Japan; 3 Faculty of Science, Shinshu University, Matsumoto, Nagano 390-8621 Japan; 4 Department of Biology and Institute of Mountain Science, Shinshu University, Matsumoto, Nagano 390-8621 Japan

**Keywords:** Behavioural ecology, Evolutionary ecology

## Abstract

Some aphid species produce a soldier caste with enlarged forelegs and horns (weapons). It has been hypothesised that the evolution of morphological specialization by soldiers in social aphids is accelerated by high predation pressure, but this possibility has not been tested. Here, we investigated the relationship between local predator abundance and soldiers’ weapon size and aggressiveness in a prey–predator system comprising a eusocial aphid, *Ceratovacuna japonica*, and its predators (larvae of the butterfly *Taraka hamada* and of the moth *Atkinsonia ignipicta*) in two populations with different predator abundances. We found that the soldiers in the predator-abundant population had larger weapons and were more aggressive than those in the population with lower predator abundance. Furthermore, the soldiers’ defensive prowess (evaluated as the survival of aphids in the presence of predators) was greater in the predator-abundant population. These results provide the first evidence that a population of eusocial aphids experiencing high predation pressure has soldiers with pronounced defensive traits and defensive prowess.

## Introduction

In social insects, morphological specialization of workers (e.g., large body, wide head) can increase the efficiency with which workers perform tasks such as colony defence, but such morphological specialization has a disadvantage that limits the task repertoire of certain workers^1–3^. For example, ants of genus *Pheidole*, have a dimorphic worker caste, with division of labour between two distinct subcastes, called major and minor^4^. Major workers are larger than minor workers and have enlarged heads and mandibular muscles. Major workers can thus generate a stronger bite force to defend against predators and competitors than minor workers can. Thus, a major worker’s task is usually limited to colony defence^5^. The morphological specialization of workers following division of labour is an important evolutionary step towards improving colony fitness in social insects^1,6^.

Some aphids, like ants, bees, wasps, and termites, exhibit sociality^7,8^. In social aphids, colony defence and cleaning are performed by certain first- or second-instar individuals; these altruistic individuals are called ‘soldiers’^7,8^. Many studies have shown that soldiers can decrease predation pressure on their colony mates (reproductives, including individuals that will potentially be able to reproduce after growth) by using their defensive traits (weapons)^7,9,10^. Soldiers show surprisingly diverse morphology among aphid taxa. Soldiers have been divided into at least four different morphological types, defined on the basis of differences in their behaviour and associated weapons^7,11^. Stern and Foster^11^ hypothesised that the evolution of soldier types occurs in five steps: (1) behavioural specialization, (2) morphological specialization, (3) prolongation of the defensive instar stage, (4) dimorphism, and (5) sterility. Therefore, in social aphids, one of the most important ecological factors in the evolution of developed sociality is likely to be natural selection for defensive behaviour and morphological traits against predators^8,12–14^. However, evidence supporting this hypothesis is lacking because it has been difficult to demonstrate a relationship between the defensive prowess of soldiers and predation pressure^8^.

In some host-alternating social aphid species, soldiers use their enlarged morphological traits (relative to those of normal individuals) such as frontal horns and forelegs for colony defence on the secondary host plant^7,8^. All social aphid species, including both those with sterile, morphologically specialised soldiers and those with fertile, non-morphologically specialised soldiers, have such traits or ‘weapons’ (e.g. *Ceratovacuna japonica*, *C. lanigera*, *Colophina clematis*, and *Pseudoregma alexanderi*)^10,15–17^. Morphologically specialised *Ceratovacuna japonica* soldiers and non-morphologically specialised *C. lanigera* soldiers show the same defensive behaviour on their secondary host plants; namely, they clutch the predator with their forelegs and pierce it with their horns^10,17^. Conspicuously, *C. japonica* soldiers have very enlarged weapons; their horn length is 1.5–1.8 times greater and their forelegs are 1.5–2.0 times longer than those of reproductive first-instar individuals^18^. Such enlargement of weapons specialised to colony defence is observed not only in aphids but also in other social insects. In termites, it has been suggested that this specialisation is selected for by high predation pressure^14,19^. Therefore, it is likely that in aphids, these enlarged weapons of soldiers are also selected for by predation pressure.

**Figure 1:**
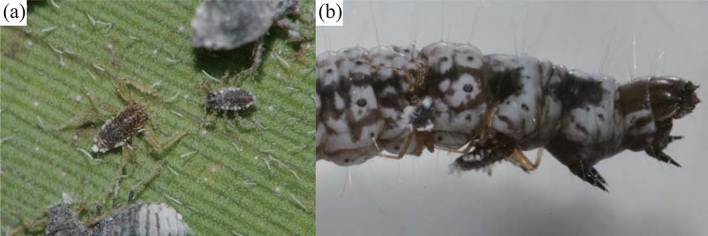
(**a**) A soldier (left) and a 1st-instar reproductive individual (right) in *Ceratovacuna japonica* (photo courtesy of Takashi Komatsu, Ibaraki Nature Museum). (**b**) Soldiers attacking a larva of *Atkinsonia ignipicta*. Soldiers attack predators with their horns and forelegs.

The eusocial aphid *Ceratovacuna japonica* produces sterile, pseudoscorpion-like soldiers on its secondary host plants, where it forms open colonies (Fig. 1a). Because of this ecological feature (open colonies), on the secondary host, we are able to measure the predation pressure and observe interactions between soldiers and predators. The soldiers attack the specialist predatory larvae of the *Taraka hamada* butterfly or *Atkinsonia ignipicta* moth using their horns and forelegs (Fig. 1b)^10^. The abundance of predators fluctuates temporally, and their numbers peak in the middle of the summer^20^. Accordingly, the size of the soldiers’ weapons and their aggressiveness also change temporally and correlate with predator abundance^20,21^. When predation risk is high, aphids produce soldiers with large weapons (forelegs and horns)^20^, and these soldiers are also more aggressive than the soldiers produced when predation risk is low^21^.

Against this background, we hypothesised that the soldiers’ weapon size and aggressiveness are related to predation pressure not only temporally^20,21^ but also spatially (over a short distance). Thus, the first aim of this study was to determine the validity of this hypothesis, for which supportive evidence was indeed obtained (see “Results”). The second aim was to clarify whether larger weapons and higher aggressiveness of soldiers actually improve their defensive prowess. By comparing the soldiers’ defensive traits (weapon size and aggressiveness) and defensive prowess (the survival of reproductives) between two populations with contrasting predator abundance, we also obtained evidence that larger weapons and greater aggressiveness do indeed aid in defence (see “Results”).

## Materials and methods

### The eusocial aphid *Ceratovacuna japonica*

*Ceratovacuna japonica* is a heteroecious (i.e. host-alternating) aphid that shows cyclic parthenogenesis characterized by several asexual phases and a sexual phase^22^. This species produces winged morphs for moving between its secondary host plant, *Sasa senanensis* (Poaceae: Bambuseae), and its primary host plant, *Styrax japonicus* (Styracaceae)^7,23^. This host-alternating behaviour can lead to clone mixing. More than five aphid clones have been found on a single leaf of *S. senanensis*^23^.

In this study, because aphids feed on the underside of *S. senanensis* leaves, we define a colony as an aggregation of aphid individuals on a single *S. senanensis* leaf.

*Ceratovacuna japonica* reproduces ‘pseudoscorpion-like’ first-instar soldiers on *S. senanensis* (Fig. 1a)^15^. Because soldiers have longer horns and forelegs than first-instar reproductive individuals^18^, we can easily distinguish between the sterile soldier caste and the reproductive caste (Fig. 1). Because soldiers never moult to the second instar, their weapon size does not change during their lifespan. The proportion of soldiers relative to the total number of individuals within a colony shows pronounced fluctuations both temporally and spatially (M. Hattori personal observation).

*Ceratovacuna japonica* is mainly preyed upon by larvae of *Atkinsonia ignipicta* (Lepidoptera: Stathmopodidae) and *Taraka hamada* (Lepidoptera: Lycaenidae), both of which are specialist predators^20,22^. These predators produce silken threads with which they make a nest on the aphid colony. When soldiers attack such a predator, they grasp its body with their forelegs and stab it with their horns. Upon being attacked by soldiers, the predator tears them off its body and bites them with its mandible^10^. Other potential predators, such as larvae of Syrphidae and Chrysophidae species, are rarely observed on aphid colonies; thus, their abundance is much lower compared with that of the specialist predators^20^.

In central Japan in particular, the main predator of *C. japonica* is *A. ignipicta* larvae. From July to August, owing to the increasing abundance of *A. ignipicta*, aphid colony size decreases by around half, and at the same time, the number of predators increases around threefold^20^. After an *A. ignipicta* larva has eaten up all of the aphids around its nest, it exits the nest and becomes free-living. As a result, many free-living *A. ignipicta* larvae can be observed on aphid colonies in August, when predation pressure peaks (M. Hattori personal observation).

### Study sites

Here, an aphid population is functionally defined as an aggregation of aphid colonies in an *S. senanensis* stand, and it can cover an area from several to 10 square metres. In this study, we focused on two populations of *C. japonica* situated at two different altitudes in Norikura, Nagano, central Japan (population A: 1452 m above sea level, 36.1220°N, 137.6288°E; population B: 1712 m above sea level, 36.1096°N, 137.6069°E). These populations were located 2.41 km apart from each other.

Population A was located at the edges of a mixed forest of *Quercus crispula* and *Betula platyphylla* with an understorey consisting of a dense stand of *S. senanensis*, the secondary host plant of *C. japonica*. This population was surrounded by roads and completely separated from populations in other *S. senanensis* stands. Population B was located at the edge of a coniferous forest of *Picea jezoensis* var. *hondoensis* with an understorey of *S. senanensis*.

### Abundance of predators

We randomly selected about 30 aphid colonies in each of the two populations and counted the number of predators found within 30 m^3^ (1.0 m depth × 20 m width × 1.5 m height) around each aphid colony from June to September 2015 (population A: June, *n* = 30, July, *n* = 30, August, *n* = 21, September, *n* = 30; population B: June, *n* = 30, July, *n* = 30, August, *n* = 30, September, *n* = 30). The predators included early-instar larvae of *A. ignipicta* and early-instar larvae of *T. hamada*, which were often found living inside silken nests^22^. To test whether the number of predators differed between the two populations in August and September, when predators are found on the colonies, we used Welch’s *t*-test with population as an independent variable.

### Weapon size of the soldiers

To examine whether the weapon size of the soldiers differed between the two populations, we measured the sizes of their horns and forelegs, because their horns and forelegs are important morphological traits used to attack their predators^10^. We randomly selected 12–17 aphid colonies in each population and selected 1–8 soldiers from each colony in each of four months. In June, July, August, and September 2015, we measured 55, 43, 65, and 75 soldiers, respectively, from population A, and 66, 54, 86 and 69 soldiers, respectively, from population B. The collected soldiers were fixed and preserved in 70% ethanol. Following the method of Kozarzhevskaya^24^, we cleared, stained, and mounted the soldier specimens on slides. We captured digitised images of the samples from their ventral side and quantified the lengths of horns and forelegs (i.e., femur length and tibia length) on the images using Photo Measure software (Kenis Ltd., Osaka, Japan) following Hattori et al.^20^. To examine whether the weapon size of the soldiers differed between the two populations in each month, we conducted nested one-way analysis of variance (ANOVA) on the weapon size, considering population as a fixed factor and colony as a random factor. Furthermore, to examine whether the weapon size varied among the months, we conducted nested one-way analysis of variance (ANOVA) on the weapon size, considering months as a fixed factor and colony as a random factor. These analyses were also followed by post hoc comparisons because we found significant population and temporal effects.

To compare the temporal change pattern between weapon size and body length in each population, we also measured the body length of the soldiers and compared body length in each population in each month. In June, July, August, and September 2015, we measured 54, 35, 65, and 74 soldiers, respectively, from population A, and 61, 52, 86 and 68 soldiers, respectively, from population B. These measured soldiers were the same as those used for the measurement of weapon size. The sample size differed between the measurement of weapon size and body length; we were not able to measure the body length of some soldiers because their bodies were bent when we made the slides. We quantified body length on the images using Photo Measure software (Kenis Ltd., Osaka, Japan) following Hattori et al.^20^. To examine whether the body length of the soldiers differed between the two populations in each month, we conducted nested one-way analysis of variance (ANOVA) on the body length, considering population as a fixed factor and colony as a random factor. Furthermore, to examine whether the body length varied among the months, we conducted nested one-way analysis of variance (ANOVA) on the body length, considering months as a fixed factor and colony as a random factor. These analyses were also followed by post hoc comparisons because we found significant population and temporal effects.

### Aggressiveness of the soldiers

We measured the duration of interaction between a soldier and a specialist predator (an *A. ignipicta* larva). We collected 17 fifth-instar larvae of *A. ignipicta* from population A on 6 August 2015. We introduced the *A. ignipicta* larvae individually into plastic Petri dishes (3.5 cm diameter) and fasted them for 24 h in an environmental chamber, which was maintained at 20 °C and a relative humidity of over 65% with a 16/8-h day/night cycle. We collected about 30 aphid colonies from each of populations A and B on 7 August 2015. The number of soldiers varied among the collected colonies. To use as many of the aphids collected in the experiment as possible, we mixed aphid individuals collected from the same population irrespective of their original colony immediately after bringing them back to our laboratory. Then, for the indoor experiment, we randomly assigned five soldiers to each experimental arena (a plastic Petri dish 3.5 cm in diameter) of two treatments: treatment 1 (*n* = 11) comprised five soldiers from population A and a predator from population A, and treatment 2 (*n* = 6) comprised five soldiers from population B and a predator from population A. When one of the soldiers attacked a predator, we removed the other four soldiers from the experimental arena and measured the duration of the interaction between the soldier and the predator. The experiments were conducted for up to 300 s. To test whether the original population of the soldiers affected the duration of interaction between a soldier and a predator, we conducted Welch’s *t*-test with the original population of the soldiers as an independent variable.

### Defensive prowess of soldiers

To examine whether their defensive prowess against a specialist predator (the *A. ignipicta* larva) varied between soldiers collected from different populations, we measured the survival rate of non-soldier aphid individuals^10^. We collected fifth-instar larvae of *A. ignipicta* from population A on 5 August 2015. We introduced the *A. ignipicta* larvae individually into plastic Petri dishes (3.5 cm diameter) and fasted them for 24 h in an environmental chamber, which was maintained at 20 °C and a relative humidity of over 65% under a 16/8-h day/night cycle. We collected about 30 aphid colonies from each of populations A and B on 6 August 2015. The numbers of soldiers and first-instar reproductive individuals varied among the collected colonies. We mixed aphid individuals collected from the same population irrespective of their original colony immediately after bringing them back to our laboratory. Then, for the indoor experiment, we randomly assigned 6 soldiers and 44 first-instar reproductive individuals to each experimental arena (a plastic Petri dish 3.5 cm in diameter) of two treatments: treatment 1 (*n* = 12) comprised 6 soldiers from population A, 44 first-instar reproductive individuals from population A, and a predator from population A; and treatment 2 (*n* = 11) comprised 6 soldiers from population B, 44 first-instar reproductive individuals from population B, and a predator from population A. Before the reproductive individuals were used in this experiment, we observed them injecting their proboscis and sucking the phloem sap from their host plant. Therefore, we consider that the behaviour of reproductive individuals used in the experiment was likely not affected by the stress of being moved from each population to the experimental arenas.

The numbers of soldier and reproductive aphids used in each treatment (6 soldiers and 44 first-instar reproductive individuals) reflects the proportion of soldiers in the natural population A; the percentage of soldiers relative to the total number of aphids in the population was 11.07 ± 2.90% (mean ± s.e.; *n* = 11). We recorded the survival rates of first-instar reproductive individuals every 30 min over a 120-min period (i.e. four times). Furthermore, we also recorded the survival rates of soldiers at 120 min. To test whether the survival rate of first-instar reproductive individuals (arcsine-transformed data) differed between populations A and B, we conducted repeated-measures ANOVA with the population as an independent variable and the observation time (30, 60, 90, 120 min) as a repeated variable. Because Mauchly’s test indicated that the sphericity assumption was violated (*χ*^2^ = 24.95, *P* < 0.003), for the repeated-measures ANOVA result we show the Greenhouse–Geisser *F* and *P* values in the “Results” section. We also used Student’s *t*-test to compare the survival rates of first-instar reproductive individuals between the populations at each timepoint.

### Statistics

We performed the statistical analyses with the JMP v. 9.0.0 statistical package (SAS Institute).

## Results

### Predator abundance

**Figure 2:**
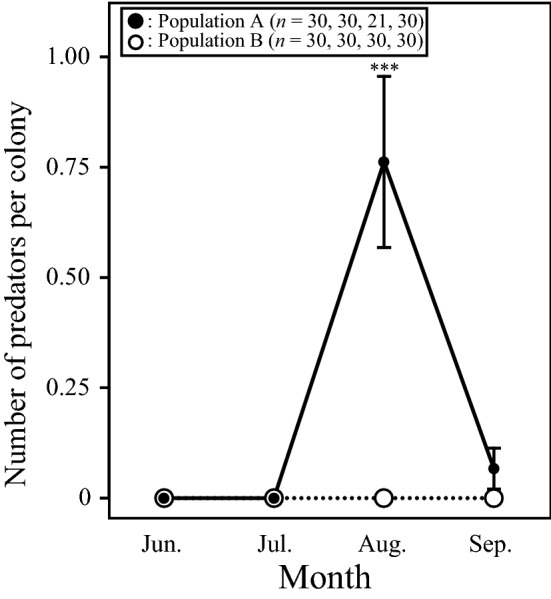
Temporal and between-population variation in the number of predators per aphid colony. The number of predators represents the sum of the numbers of *A. ignipicta* and *T. hamada* larvae. Error bars denote s.e. Asterisks indicate a significant difference between populations (***P < 0.001).

The number of predators on the aphid colonies varied spatiotemporally (Fig. 2). In particular, the number of predators in population A was significantly larger than that in population B in August but not in September (August, *t*_20_ = 3.93, *P* < 0.001; September, *t*_29_ = 1.44, *P* > 0.05). In population A, we found predators on the aphid colonies in August and September, but not in June and July. In August, the only predators found were *A. ignipicta* larvae (0.76 ± 0.19 individuals per aphid colony), whereas in September the predators comprised both *A. ignipicta* larvae (0.033 ± 0.033 individuals per aphid colony) and *T. hamada* larvae (0.033 ± 0.033 individuals per aphid colony). In population B, we found no predators in any of the months.

### Soldier weapon size

**Figure 3:**
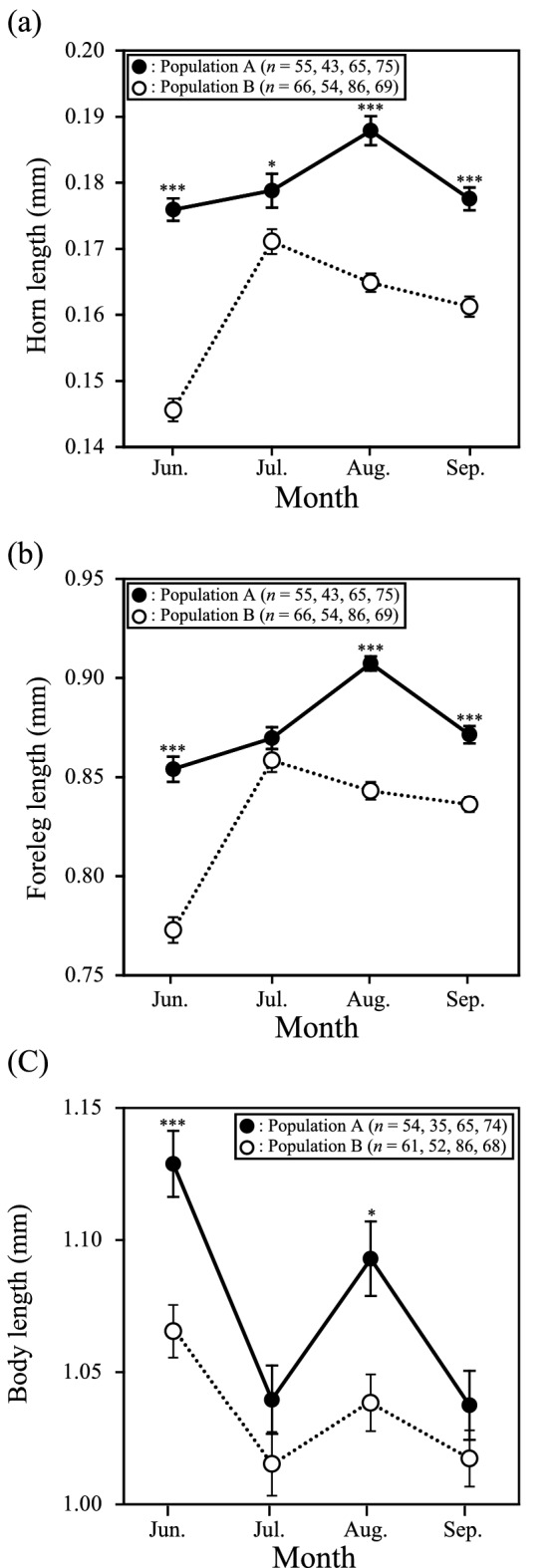
Temporal and between-population variation in the weapon size of soldiers. The average (**a**) horn, (**b**) foreleg, and (**c**) body length of soldiers in populations A and B from June to September are shown. Error bars denote s.e. Asterisks indicate significant differences between populations (*P < 0.05; ***P < 0.001).

The horns of soldiers from population A were longer than those of soldiers from population B in all months (June, *F*_1,29_ = 84.45, *P* < 0.001; July, *F*_1,24_ = 5.26, *P* = 0.030; August, *F*_1,27_ = 31.34, *P* < 0.001; September, *F*_1,26_ = 34.16, *P* < 0.001; Fig. 3a). Forelegs of soldiers from population A were longer than those of soldiers from population B in three of the months, with the exception of July (June, *F*_1,29_ = 55.32, *P* < 0.001; July, *F*_1,24_ = 0.56, *P* = 0.46; August, *F*_1,27_ = 88.29, *P* < 0.001; September, *F*_1,26_ = 20.72, *P* < 0.001; Fig. 3b).

Furthermore, in population A, horn length varied temporally (*F*_3,52_ = 3.96, *P* = 0.012). Post hoc comparisons revealed that horn length was significantly longer in August than in June or September (June vs. August, *F*_1,27_ = 7.96, *P* = 0.008, August vs. September, *F*_1,27_ = 6.07, *P* = 0.020; Fig. 3a), but did it not vary between other months (June vs. September, *F*_1,28_ = 0.52, *P* > 0.05, July vs. August, *F*_1,21_ = 0.98, *P* > 0.05, July vs. September, *F*_1,22_ = 0.06, *P* > 0.05; Fig. 3a).

In addition, in population A, foreleg length varied temporally (*F*_3,52_ = 12.67, *P* < 0.001). Post hoc comparisons revealed that foreleg length was significantly longer in August than in the other 3 months (June vs. August, *F*_1,27_ = 37.09, *P* < 0.001, July vs. August, *F*_1,21_ = 15.32, *P* < 0.001, August vs. September, *F*_1,27_ = 25.86, *P* < 0.001; Fig. 3b), but it did not vary between other months (June vs. July, *F*_1,25_ = 1.00, *P* > 0.05, June vs. September, *F*_1,28_ = 1.80, *P* > 0.05, July vs. September, *F*_1,22_ = 0.06, *P* > 0.05; Fig. 3b).

In population B, horn length and foreleg length varied temporally (horn length, *F*_3,56_ = 26.29, *P* < 0.001; foreleg length, *F*_3,56_ = 31.49, *P* < 0.001). Post hoc comparisons revealed that horn length in June was significantly shorter than in the other months (June vs. July, *F*_1,28_ = 67.21, *P* < 0.001, June vs. August, *F*_1,29_ = 42.76, *P* < 0.001, June vs. September, *F*_1,29_ = 29.48, *P* < 0.001; Fig. 3a) and horn length was significantly longer in July than in the other months (July vs. August, *F*_1,27_ = 6.05, *P* = 0.020, July vs. September, *F*_1,27_ = 13.74, *P* < 0.001; Fig. 3a), whereas horn length in August was not significantly different from that in September (August vs. September, *F*_1,28_ = 1.66, *P* > 0.05; Fig. 3a).

Furthermore, post hoc comparisons revealed that foreleg length was significantly shorter in June than in the other months (June vs. July, *F*_1,28_ = 50.71, *P* < 0.001, June vs. August, *F*_1,29_ = 42.05, *P* < 0.001, June vs. September, *F*_1,29_ = 43.54, *P* < 0.001; Fig. 3b), and foreleg length was significantly longer in July than in the other months (July vs. August, *F*_1,27_ = 4.64, *P* = 0.039, July vs. September, *F*_1,27_ = 8.30, *P* = 0.007; Fig. 3b), but foreleg length in August was not significantly different from that in September (August vs. September, *F*_1,28_ = 0.63, *P* > 0.05; Fig. 3b).

The body length of soldiers from population A was greater than that of soldiers from population B in June and August (June, *F*_1,29_ = 22.02, *P* < 0.001; July, *F*_1,23_ = 1.57, *P* > 0.05; August, *F*_1,27_ = 7.15, *P* = 0.015; September, *F*_1,28_ = 1.40, *P* > 0.05; Fig. 3c).

Furthermore, in population A, body length varied temporally (*F*_3,51_ = 9.91, *P* < 0.001). Post hoc comparisons revealed that body length was significantly longer in August than in July or September (July vs. August, *F*_1,23_ = 4.64, *P* = 0.04, August vs. September, *F*_1,27_ = 7.32, *P* = 0.01; Fig. 3c) and it was longer in June than in July or September (June vs. July, *F*_1,28_ = 10.59, *P* = 0.002, June vs September, *F*_1,29_ = 10.66, *P* = 0.002; Fig. 3c). However, it did not vary between other months (June vs. August, *F*_1,29_ = 2.87, *P* > 0.05, July vs. September, *F*_1,24_ = 0.02, *P* > 0.05; Fig. 3c). Therefore, in population A, the soldiers’ weapon size, which was largest in August, was exaggerated in that month in comparison with body length because their body length was not greatest in August.

In population B, body length also varied temporally (*F*_3,56_ = 2.79, *P* = 0.05). Post hoc comparisons revealed that body length was significantly longer in June than in July or September (June vs. July, *F*_1,24_ = 21.21, *P* < 0.001, June vs September, *F*_1,28_ = 27.18, *P* < 0.001; Fig. 3c). However, it did not vary between other months (June vs. August, *F*_1,27_ = 3.74, *P* > 0.05, July vs. August, *F*_1,27_ = 0.72, *P* > 0.05, July vs. September, *F*_1,27_ = 0.02, *P* > 0.05, August vs. September, *F*_1,28_ = 0.61, *P* > 0.05; Fig. 3c). Therefore, in population B, the soldiers’ weapon size was exaggerated compared to their body length in August because their body length was not greatest in August.

### Soldier aggressiveness

**Figure 4:**
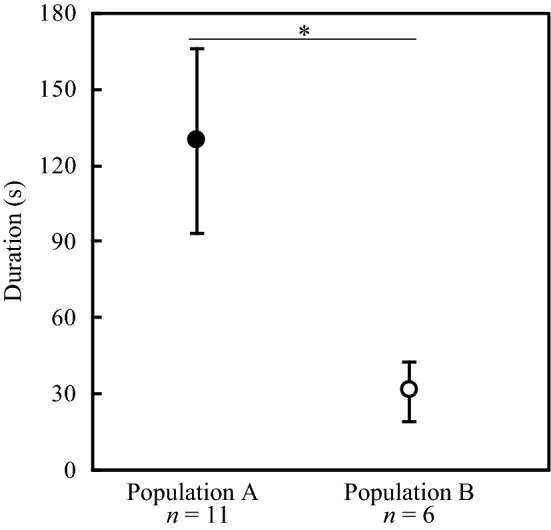
Duration of aggressive interaction between a soldier and a specialist predator (*A. ignipicta* larva) in the two populations. The duration from when the soldier first grasped the predator until the predator tore the soldier off its body was measured in a Petri dish (population A: *n* = 6, population B: *n* = 11). The soldiers from population A (filled circle), which had a higher predator density in the wild and soldiers with larger weapons and higher aggressiveness, interacted with the predator for significantly longer than those from population B (open circle), which had a lower predator density in the wild (Welch’s *t*-test). Symbols and error bars indicate mean ± s.e. The asterisks indicate a significant difference between populations (*P < 0.05).

When encountering a predatory *A. ignipicta* larva, a soldier immediately grasped the predator and pierced it with the horns. In response, the predator attempted to tear the soldier off by biting or pulling at it with the mandibles. Once the soldier was repelled, it immediately became motionless and did not fight again. The duration of this aggressive interaction between a soldier and the predator differed between the populations (*t*_11.91_ = 2.58, *P* = 0.024; Fig. 4). The predator needed four times longer to tear a soldier from population A off its body than one from population B [population A (*n* = 11): 129.54 ± 36.35 s (mean ± s.e.); population B (*n* = 6): 30.94 ± 11.67 s (mean ± s.e.)]. No soldier killed the predator during the observation period in any of the treatments of this experiment.

### Soldier defensive prowess

**Figure 5:**
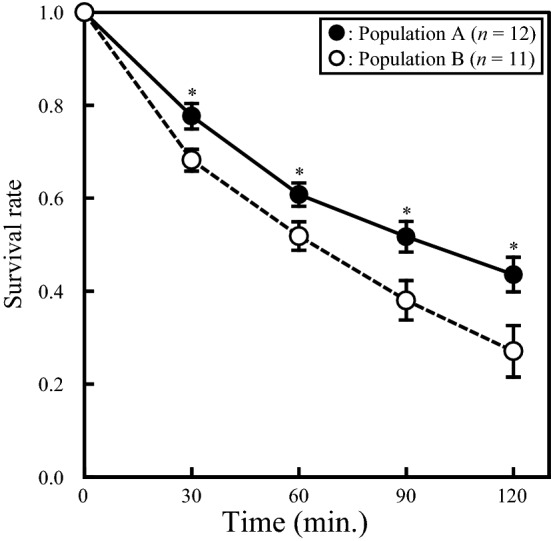
The defensive prowess of soldiers against predatory *A. ignipicta* larvae. The survival rate of 44 first-instar reproductive individuals introduced into a Petri dish with six soldier aphids and a starved predator was monitored for 2 h. Soldiers from population A were used in 12 Petri dishes, and soldiers from population B were used in 11 Petri dishes. The first-instar reproductive individuals protected by soldiers from population A (filled circle), which had a higher predator density in the wild and soldiers with larger weapons and higher aggressiveness, had a significantly higher survival rate than those collected from population B (open circle), which had a lower predator density in the wild (repeated-measures ANOVA). Symbols and error bars indicate mean ± s.e. Asterisks indicate significant differences between populations (*P < 0.05).

In both treatments, the survival rate of first-instar reproductive individuals gradually decreased with time, but the pattern of decrease differed between the populations (repeated-measures ANOVA, *F*_2.36,49.57_ = 3.75, *P* = 0.024; Fig. 5). The survival rate of the first-instar reproductive individuals at 120 min was 44% in population A and 27% in population B. These results indicate that soldiers from population A improved the survival rate of first-instar reproductive individuals 1.5-fold compared with soldiers from population B. The survival rate of the soldiers at 120 min was 24% in population A and 17% in population B. The soldiers that did not survive were killed by the predator. No soldier killed the predator during the observation period in any of the treatments of this experiment.

The difference in the survival rate of first-instar reproductive individuals between the treatments was significant at all observation times (30 min: *t*_21_ = 2.70, *P* = 0.014; 60 min: *t*_21_ = 2.25, *P* = 0.036; 90 min: *t*_21_ = 2.57, *P* = 0.019; 120 min: *t*_21_ = 2.56, *P* = 0.020). Hence, the survival rate of first-instar reproductive individuals differed between the populations soon after the beginning of the experiment, and the difference became more pronounced over time.

## Discussion

In eusocial aphids that produce a sterile soldier caste, the relationship between the soldiers’ defensive prowess and predation pressure has not been tested previously. Our results showed that in the eusocial aphid *C. japonica*, the soldiers’ weapon size, aggressiveness against specialist predators, and defensive prowess differed between populations. Our results also revealed that these differences positively correlated with the geographical variation of predation pressure.

### Correlation between defensive prowess and soldier phenotype

The defensive prowess of soldiers, reflected in the survival rate of first-instar reproductive individuals in our experiment (Fig. 5), was higher in population A, which had better armed and more aggressive soldiers, than in population B. Because first-instar reproductive individuals are unable to defend themselves against predators^10^, the higher survival rate in population A appears to have been caused by the soldiers’ defensive abilities, which are affected by two traits. One is their weapon size; the soldiers in population A had longer weapons than those in population B (Fig. 3). The other is their aggressiveness; our results showed that the soldiers in population A carried out biting attacks for longer than those in population B (Fig. 4). These results show that the soldiers’ prowess was related to their weapon size and aggressiveness.

### Influence of predation pressure on the morphological specialization in soldiers

Our results showed spatial variation in the defensive performance of soldiers between populations located not very far apart. This variation might be due differences in the clone characteristics of each population. However, local differences in the selection pressure on the defensive traits of soldiers appear to be more important. Because of the host-alternating behaviour of the aphids, a population can be supplied with new clones every year. As a result, it may undergo selection repeatedly in response to predation pressure. Indeed, Hattori et al.^23^ have shown that a colony consists of multiple clones. Adaptive defensive traits of soldiers should differ between populations with low and high predation pressure. The frequency of aggressive clones may decrease in a population with low predation pressure because the investment in soldiers with large weapons may be more energetically and developmentally expensive for the reproductive individuals than that in soldiers with small weapons. Because each population may face predation pressure at different intensities, the soldiers may exhibit variation in their defensive traits.

This proposed scenario does not contradict the prediction of previous studies that the defensive traits of soldiers are the direct or indirect target of natural selection from predation^7,8^. Variation in predation pressure leads to variation in the investment in soldiers because producing sterile soldiers is costly for colony growth. There are two ways to invest in soldiers. The first way is to increase the number of soldiers produced. Increasing the soldier birth rate in a colony, so that proportion of soldiers relative to reproductives is increased, can mitigate predation pressure on the colony. The second way is to improve the soldiers' effectiveness, that is, the ability of the soldiers that are produced to defend the colony (e.g., by producing soldiers with larger forelegs and higher aggressiveness). Although increasing the investment in soldiers by changing number of soldiers produced has been reported frequently^13,26,27^, improvement in the effectiveness of soldiers has been reported more rarely (but see^20,21^). Because the efficiency of the defensive strategy of social aphids depends on the balance between the number of soldiers and their effectiveness, further study of the relationship between a soldier’s phenotypic traits and its defensive abilities is needed to further our understanding of the defensive strategy of social aphids.

Several eusocial aphid species (e.g. *Pseudoregma bambusicola, P. sundanica, C. japonica*) produce morphologically specialised sterile individuals (a sterile soldier caste) that are specialised for colony defence^7^. In particular, Cerataphidini aphids, including *C. japonica*, have evolved soldiers that use their horns for colony defence on their secondary host^7^. Previous studies have suggested that this defensive behaviour using horns originates from conspecific competition for feeding sites^7,25^. In *Astegopteryx bambucifoliae*, individuals attack other feeding conspecific individuals by butting them with their horns^25^. This behaviour is very similar to the defensive behaviour of soldiers against larvae and eggs of predators in *C. lanigera* and *C. japonica* (Fig. 1)^10,17^. However, in this study of *C. japonica*, defensive phenotype and defensive prowess both correlated with predation pressure. This result suggests that *C. japonica* soldiers with enlarged weapons exhibit greater defensive prowess than *C. lanigera* soldiers with non-enlarged weapons. Therefore, in *C. japonica*, the enlarged weapons of soldiers may have evolved under selection from higher predation pressure. A future study should compare interactions between aphids and predators among *Ceratovacuna* species on secondary hosts (e.g. among *C. japonica*, which produces enlarged sterile soldiers, *C. lanigera*, which produces smaller non-sterile soldiers, and *C. nekoashi*, which does not produce soldiers).

### Induced defence of *Ceratovacuna japonica*

Plasticity of the defensive response of prey has been termed ‘induced defence’^28–31^. Shingleton and Foster^27^ showed that soldiers are induced in the eusocial aphid *P. sundanica* when the aphids are not tended by ants. Investment in soldiers controlled by an induced defence is a good answer to a predation risk that changes spatially and temporally. In *C. japonica*, Hattori et al.^20^ showed that the temporal variation in the weapon size of soldiers is partly caused by a plastic maternal response to predation risk. Maternal aphids reared at 20 °C (the monthly average temperature in central Japan in midsummer when predators are abundant^20^) produced soldiers with larger weapons more frequently than those reared at 15 °C [the monthly average temperature in central Japan in early summer when predators are less abundant (c.f.,^20^)]. Similarly, in this study, the soldiers’ weapon size was larger when predators were abundant (i.e. in August) than in other months (i.e. in June and September) in population A (Fig. 2). Furthermore, in August, soldiers from population A were more aggressive and showed greater defensive prowess than those from population B (Figs. 4 and 5). Accordingly, temporal variation in the weapon size of soldiers may be related to their having greater aggressiveness and defensive prowess. Our results showed that *C. japonica* responds to the temporal variation of predation risk by the plastic change of soldiers’ defensive prowess as an induced defence.

However, our results also showed that the weapon size of the soldiers is not fully controlled by a plastic maternal response to temperature because, although the body length of soldiers did not be influenced by a plastic maternal response^20^, the body length of soldiers significantly varied temporally in both populations (Fig. 3c). Therefore, under natural conditions, various factors (e.g., competition between aphid clones and phenotypic selection by predators) can affect the weapon size of the soldiers. Many prey species show an induced defence against direct predation risk (e.g., the presence of predators, predation by conspecific individuals)^27–30^, and *C. japonica* may also change the weapon size of the soldiers produced in the face of not only indirect predation risk (i.e., temperature) but also direct predation risk. This scenario is not inconsistent with the temporal pattern of change in the soldiers’ weapon size in population B (Fig. 3a,b). In population B, maternal aphids may have stopped producing soldiers with large weapon sizes in an environment without predators. Further study is needed to reveal all of the factors affecting the weapon size of soldiers under natural conditions.

## Conclusions

Stern et al.^32^ suggested that soldier morphology in aphids is responsive to selective pressure from predators, but this possibility has not previously been tested. Here, we focused on the relationship between the defensive prowess of soldiers and predation pressure. Our results showed that in *C. japonica*, the weapon size, aggressiveness against a specialist predator (*A. ignipicta*), and defensive prowess of soldiers differed between populations. Our results also revealed that these differences correlated with geographical variation of predation pressure. Therefore, this study provides the first evidence that in aphids, high predation pressure can lead to the evolution of morphological specialization in soldiers. Further studies are needed to reveal whether the defensive prowess of soldiers is related to predation pressure in other social aphid species. Such studies would deepen our understanding of the evolution of the soldier caste in aphids.

## Supplementary Information

Supplementary Figure S1.

Supplementary Information 2.

## Data Availability

All data analyzed during this study are included a Supplementary material file.
