# Supplementary material for: Positive geographic correlation between soldiers’ weapon size and defensive prowess in a eusocial aphid, Ceratovacuna japonica
Source: Sci Rep. 2022 Sep 23;12:15874. doi: 10.1038/s41598-022-20389-z (PMC9508264; doi:10.1038/s41598-022-20389-z)
Supplement: Supplementary file 1 — Supplementary Figure S1. [file 41598_2022_20389_MOESM1_ESM.docx]

**Supplementary Information**


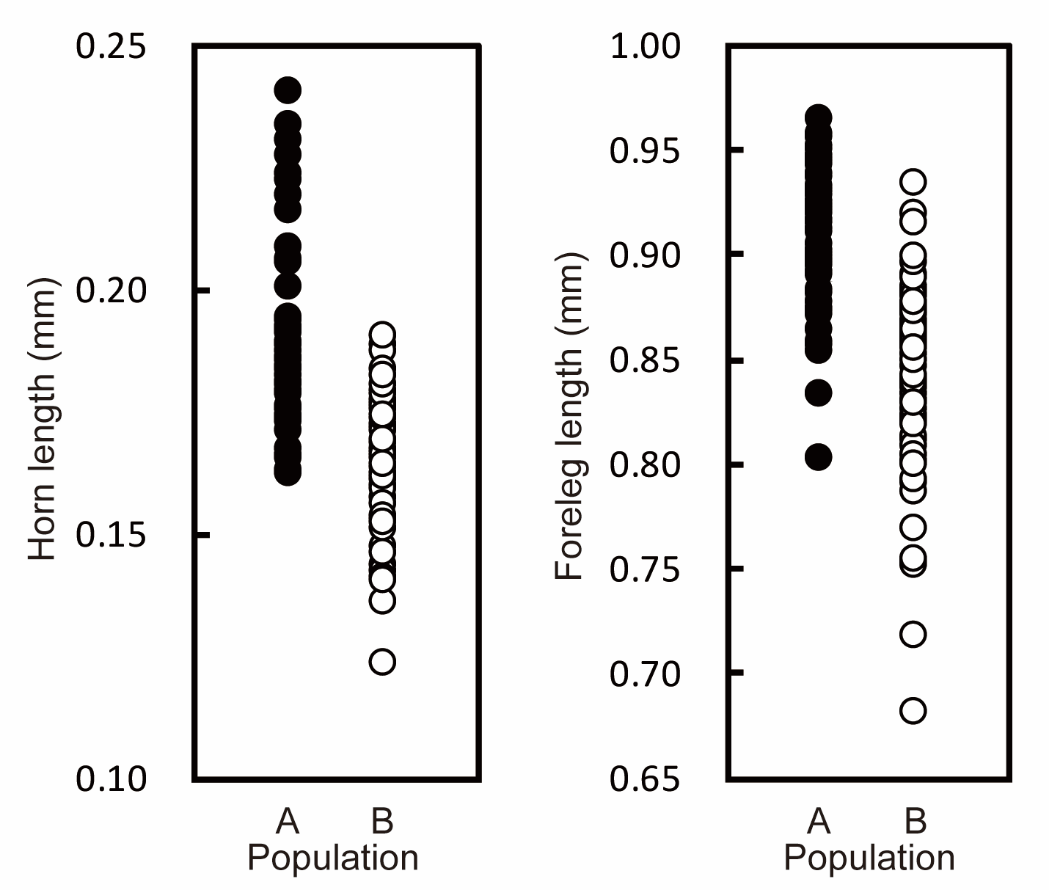


**Fig. S1** Variation of weapon size of soldiers between populations in August. Each individual point shows the weapon size of each soldier. The soldiers that were collected from population A (●, higher predator density in the wild, soldiers with larger morphological traits and higher aggressiveness) interacted with the predator for significantly longer than those collected from population B (○, lower predator density in the wild).
